# Pure Homœopathy *vs.* Eclecticism

**Published:** 1895-01

**Authors:** Jos. Fitz-Mathew

**Affiliations:** Victoria, Dauphin Co., Pa.


### PURE HOMŒOPATHY *vs.* ECLECTICISM.

JOS. FITZ-MATHEW, M. D., VICTORIA, DAUPHIN CO., PA.

When some years ago resolutions were introduced in the New York Homœopathic Medical Society, which, if not rescinded, would have practically converted it into an Eclectic Society, our enemies were jubilant. They said what they wished, that Homœopathy was moribund. But if we consider the number of practitioners of Homœopathy which have since been launched they have a very lively corpse to deal with.

Even if a majority of these practitioners do mix up Eclecticism with Pure Homœopathy, *they do not bring Homœopathy down to their level,* nor does it affect the standing of Homœopathy in the opinions of competent judges.

It is certain that now a large proportion of homœopaths do—occasionally, at any rate—resort to eclectic remedies. But "why do they persist in sailing under the flag of Homœopathy"? It is because they know that a homœopathic practitioner enjoys a superior professional standing in public estimation. It takes the *name* of Homœopathy to make their eclectic remedies "go down" with patients who seek a homœopath.

It seems to me entirely unnecessary to attempt to prove in these days that the principles and practice of Homœopathy, being founded upon a "Rock of Truth," can never die. The practice of those worthy disciples of the great master and founder of Homœopathy, many of whom have grown gray in the service among us, could produce an amount of evidence which would be irrefutable. But, "why have we so many backsliders amongst us"? I divide them into two classes, viz., those who prescribe according to similia as long as no case presents itself in which they fail to find a remedy to cover the symptoms, and then, harassed by the importunities of a suffering patient, who wants relief from present pain, and by the fear of dismissal if they do not afford it, they resort to a palliative. The second class comprises those who are habitually eclectic in their practice. They have, some of them, a very liberal bill of fare for their patients. "Take your choice, gentlemen." You can have Allopathy, Homœopathy, or Eclectic treatment out of the same bottle. It is a pity Heller did not study medicine. He might have beaten them at their own game.

Now, I sympathize with some of the first class. Cases occur in which it requires great labor and mental effort to arrange and to elicit the patient's symptoms and to find a remedy which fits the case, and in desperation they depart from Homœopathy. *Inadequate medical education, especially* in materia medica, is the root of this evil. Supposing a case presented itself for the symptoms of which no remedy was to be found, after every effort, which coincided with the presented symptoms, and in an emergency some measure of palliation is adopted! This is no discredit to Homœopathy, for we have yet to discover, prove, and record many more drugs which may supply deficiencies in our materia medica. It is a good legal axiom that "every case must stand upon its own legs." I don't believe that any homœopathist who has common sense, and who has repeatedly seen the beneficent action of our well-prescribed remedies, would ever resort to other measures within the range of medicine if he could find the simillimum. Those who have had the experience of years of practice well know the labor encountered in finding a remedy in a given case. They also know that a patient who will be patient in the hands of a renowned practitioner will often not be so in those of his less fortunate brethren, to whom the loss of a patient and the chagrin of a failure is much more serious. But I believe many of these straying homœopathists will come back "into the fold" of their own accord as they gain in experience and the knowledge of materia medica, and realize the failure which inevitably follows every departure from *similia similibus curantur.*

